# Metagenomic next-generation sequencing confirms the diagnosis of *Legionella* pneumonia with rhabdomyolysis and acute kidney injury in a limited resource area: a case report and review

**DOI:** 10.3389/fpubh.2023.1145733

**Published:** 2023-05-09

**Authors:** Rao Du, Yinhe Feng, Yubin Wang, Jifeng Huang, Yuhan Tao, Hui Mao

**Affiliations:** ^1^Department of Respiratory and Critical Care Medicine, West China Hospital, Sichuan University, Chengdu, China; ^2^Department of Respiratory and Critical Care Medicine, Deyang People's Hospital, Affiliated Hospital of Chengdu College of Medicine, Deyang, China

**Keywords:** *Legionella* pneumonia, rhabdomyolysis, acute kidney injury, metagenomic next-generation sequencing, case report

## Abstract

**Background:**

*Legionella* pneumonia, rhabdomyolysis, and acute kidney injury are called the *Legionella* triad, which is rare and associated with a poor outcome and even death. Early diagnosis and timely treatment are essential for these patients.

**Case presentation:**

A 63-year-old man with cough, fever, and fatigue was initially misdiagnosed with common bacterial infection and given beta-lactam monotherapy but failed to respond to it. Conventional methods, including the first *Legionella* antibody test, sputum smear, and culture of sputum, blood, and bronchoalveolar lavage fluid (BALF) were negative. He was ultimately diagnosed with a severe infection of *Legionella pneumophila* by metagenomics next-generation sequencing (mNGS). This patient, who had multisystem involvement and manifested with the rare triad of *Legionella* pneumonia, rhabdomyolysis, and acute kidney injury, finally improved after combined treatment with moxifloxacin, continuous renal replacement therapy, and liver protection therapy.

**Conclusion:**

Our results showed the necessity of early diagnosis of pathogens in severe patients, especially in Legionnaires' disease, who manifested with the triad of *Legionella* pneumonia, rhabdomyolysis, and acute kidney injury. mNGS may be a useful tool for Legionnaires' disease in limited resource areas where urine antigen tests are not available.

## Introduction

*Legionella pneumophila* (*L. pneumophila*), a species of the *Legionella* genus, is the causative agent of Legionellosis, which contains two forms: the non-pneumonic form (Pontiac fever) and the acute pneumonic form (Legionnaires' disease) (1). Pontiac fever is an influenza-like syndrome. Legionnaires' disease is more severe and can be involved in extrapulmonary manifestation with severe pneumonia, which requires hospitalization and most commonly intensive care. The mortality was 4.6% in medical wards compared with 23.1% in the intensive care unit (ICU) (2). Extrapulmonary manifestations included abdominal pain, diarrhea of the digestive system, weakness, and fatigue of the musculoskeletal system, myoglobinuria of the urinary system, malaise of the nervous system, etc. The triad of *Legionella* pneumonia, rhabdomyolysis, and acute kidney injury (AKI) was rare, and its mortality was much higher than in patients who manifested only with *Legionella* pneumonia. Early diagnosis and prompt treatment is critical for such patients. However, the existing diagnosis method of legionellosis cannot meet the clinical demands.

Metagenomic next-generation sequencing (mNGS), a culture-independent method, can detect all pathogens from one specimen (3) and has been recommended by expert consensus for diagnosing challenging cases of complicated infectious disease (4). It is especially suitable for suspected infectious diseases with negative conventional methods. Herein, we present a case of *Legionella pneumophilia* infection that manifested initially as cough, fever, and fatigue, and led to a rare triad of *Legionella* pneumonia, rhabdomyolysis, and AKI, which was ultimately diagnosed by mNGS.

## Case presentation

**Figure 1:**
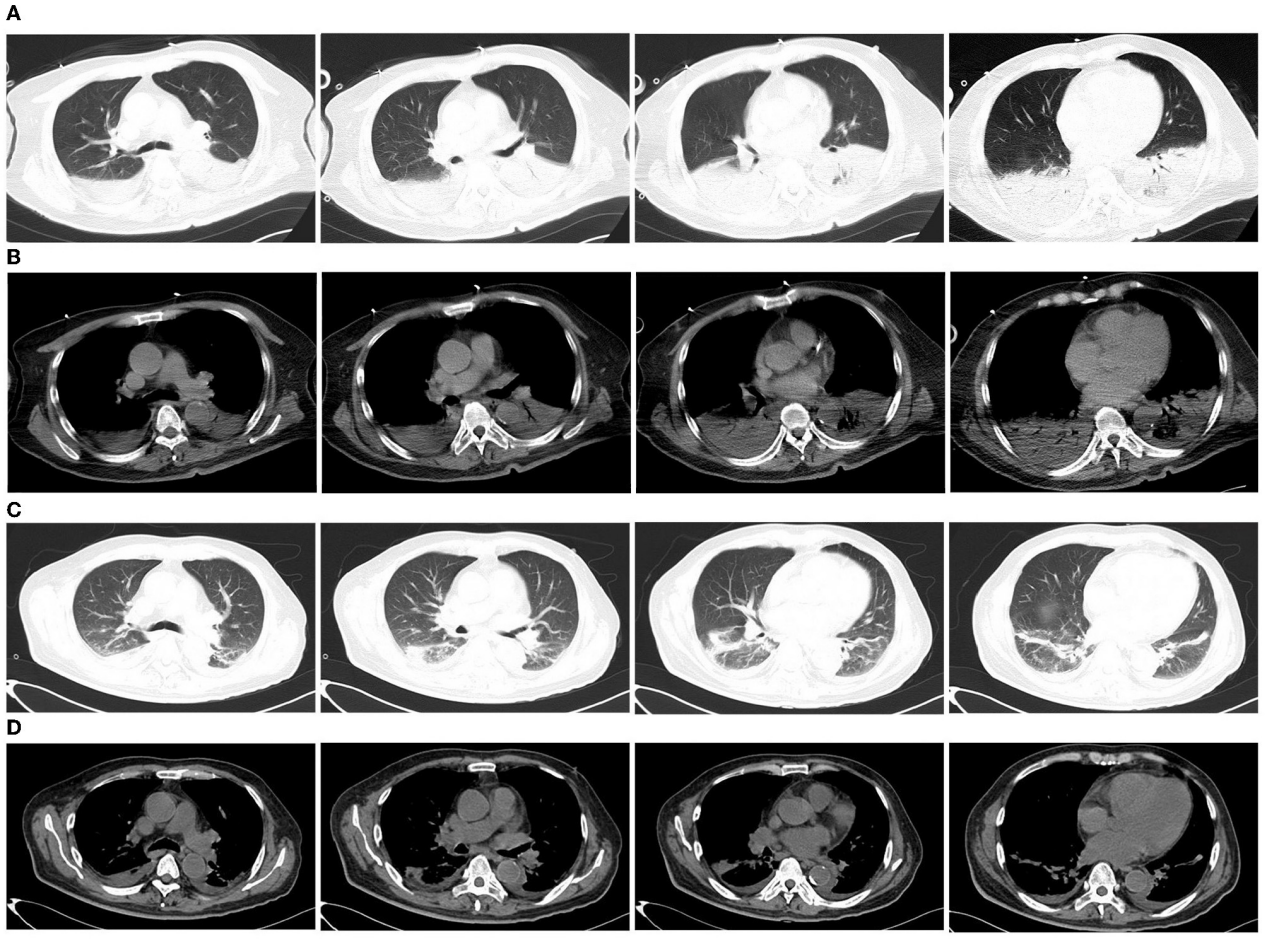
Chest computed tomography (CT) of the patient. **(A, B)** Chest CT on the first day of admission to the emergency department showed consolidation of the lower lobes of both lungs; **(C, D)** Chest CT 23 days after moxifloxacin treatment showed that the lesions were significantly absorbed.

A 63-year-old man presented to the emergency department with a 3-day history of cough, fever, and fatigue. He was receiving piperacillin-tazobactam monotherapy at a local hospital. However, the patient failed to respond to the treatment. Initial vital signs were body temperature (>40°C), pulse 148 beats per minute, and blood pressure 146/73 mmHg. Although he had been administered 10 L of oxygen via a nasal cannula, the peripheral oxygen saturation was only 87%. Physical examination revealed somnolent consciousness, and auscultation revealed decreased breath sounds and scattered rales in both lower lobes. The muscle strength of the upper limbs was grade four, and that of the lower limbs was grade two. The initial laboratory test results are presented in Table 1. Urine analysis showed hematuria. The first serum antibody test of anti-*Legionella* was negative. DNA tests for chlamydia and mycoplasma were negative, as well as viral pharyngeal swabs for influenza A and B. Electrocardiography revealed sinus tachycardia. Computed tomography (CT) revealed extensive consolidation in both the lower lobes (Figures 1A, B). Invasive intubation and continuous renal replacement therapy (CRRT) were initiated immediately after admission, and intravenous meropenem (1,000 mg q8h) was given on the first day. Given that the patient had confusion, hyponatremia, elevated creatine kinase (CK), and severe pneumonia, Legionnaire's disease was suspected. Thus, moxifloxacin (400 mg, once a day) was added the next day for treatment. He was admitted to the intensive care unit (ICU) for the management of acute respiratory failure, massive rhabdomyolysis, and AKI.

**Table 1:** Laboratory analysis at admission.

**Laboratory analysis**	**Level**	**Normal range**
WBC	11.65 × 10^9^/L	3–10 × 10^9^/L
NEU%	94.0%	40–75%
PCT	>100 ng/ml	< 0.046 ng/ml
CRP	295.00 mg/L	< 5 mg/L
IL-6	111.00 pg/ml	0–7.00 pg/ml
ALT	173 IU/L	< 40 IU/L
AST	571 IU/L	< 35 IU/L
LDH	1,213 IU/L	120–250 IU/L
CK	27,848 IU/L	20–140 IU/L
Myoglobin	>3000.00 ng/mL	< 58.0 ng/mL
CK-MB	25.12 ng/mL	< 2.88 ng/mL
TPN-T	73.0 ng/L	0–14 ng/L
serum creatinine	616.00 μmol/L	48–79 μmol/L
BUN	16.5 mmol/L	2.6–7.5 mmol/L
eGFR	19.70 ml/min/1.73m^2^	>90 ml/min/1.73m^2^
Na^+^	130.9 mmol/L	137.0–147.0 mmol/L
Glucose	24.0 mmol/L	3.90–5.90 mmol/L
Glycosylated hemoglobin	13.3%	< 6.0%

WBC, white blood cell; NEU, neutrophil; ALT, alanine transaminase; AST, aspartate transaminase; LDH, lactate dehydrogenase; CK, creatine kinase; TPN-T, troponin T; BUN, blood urea nitrogen; eGFR, estimated glomerular filtration rate; Na, sodium.

## Diagnostic assessment

The patient's sputum smear and sputum, blood, and bronchoalveolar lavage fluid (BALF) conventional common bacteria cultures failed to reveal a pathogen. Therefore, BALF and lung tissue samples were sent for mNGS analysis (BGI-500, Chengdu, China). The BGI company conducted mNGS as described previously (5), using samples of 0.5–3 mL sputum or lung tissue collected following standard procedures. The sample was agitated at 2,800–3,200 rpm for 30 min in a 1.5 mL microcentrifuge tube. DNA was extracted from a new tube of 0.3 mL sample tube using the TIANamp Micro DNA Kit (DP316; Tiangen Biotech, http://tiangen.com) according to the manufacturer's recommendations. DNA libraries were constructed using DNA fragmentation, end repair, adapter ligation, and PCR amplification. It generated high-quality sequencing data from sequencing libraries using the BGISEQ-500 platform. Subsequently, computational subtraction of the human host sequences (hg19) was performed and low-quality and short reads (< 50 bp) were removed by Burrows-Wheeler Alignment, as well as low-complexity reads, the remaining data were classified by aligning them to BGI self-established database downloaded from NCBI (ftp://ftp.ncbi.nlm.nih.gov/genomes/), which contains 1,798 whole genome sequences of DNA viral taxa, 6,350 bacterial genomes or scaffolds, 1,064 fungi related to human infection, and 234 parasites associated with human diseases.

**Figure 2:**
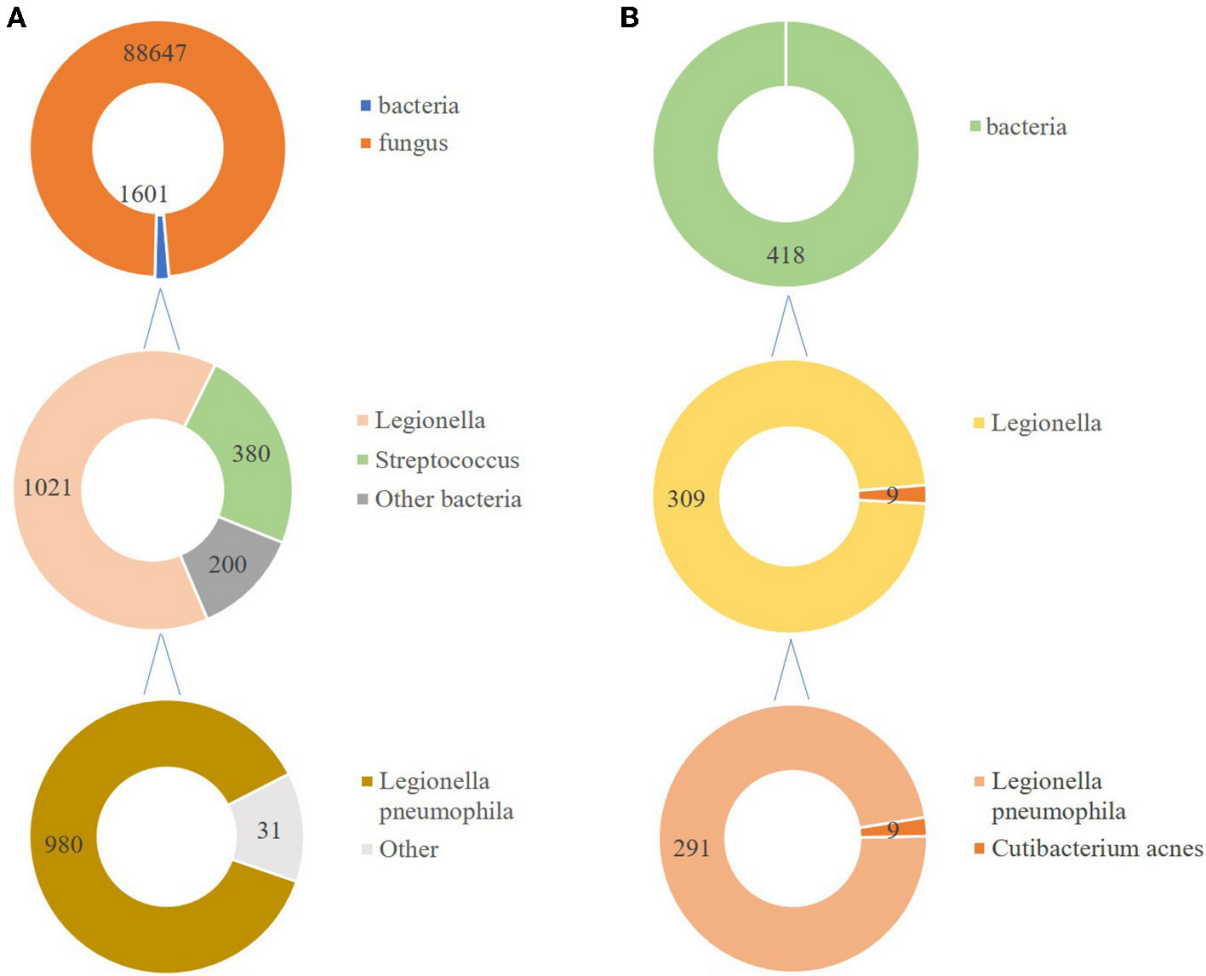
The corresponding reads of detected microorganism in **(A)** BALF and **(B)** lung tissue.

**Figure 3:**
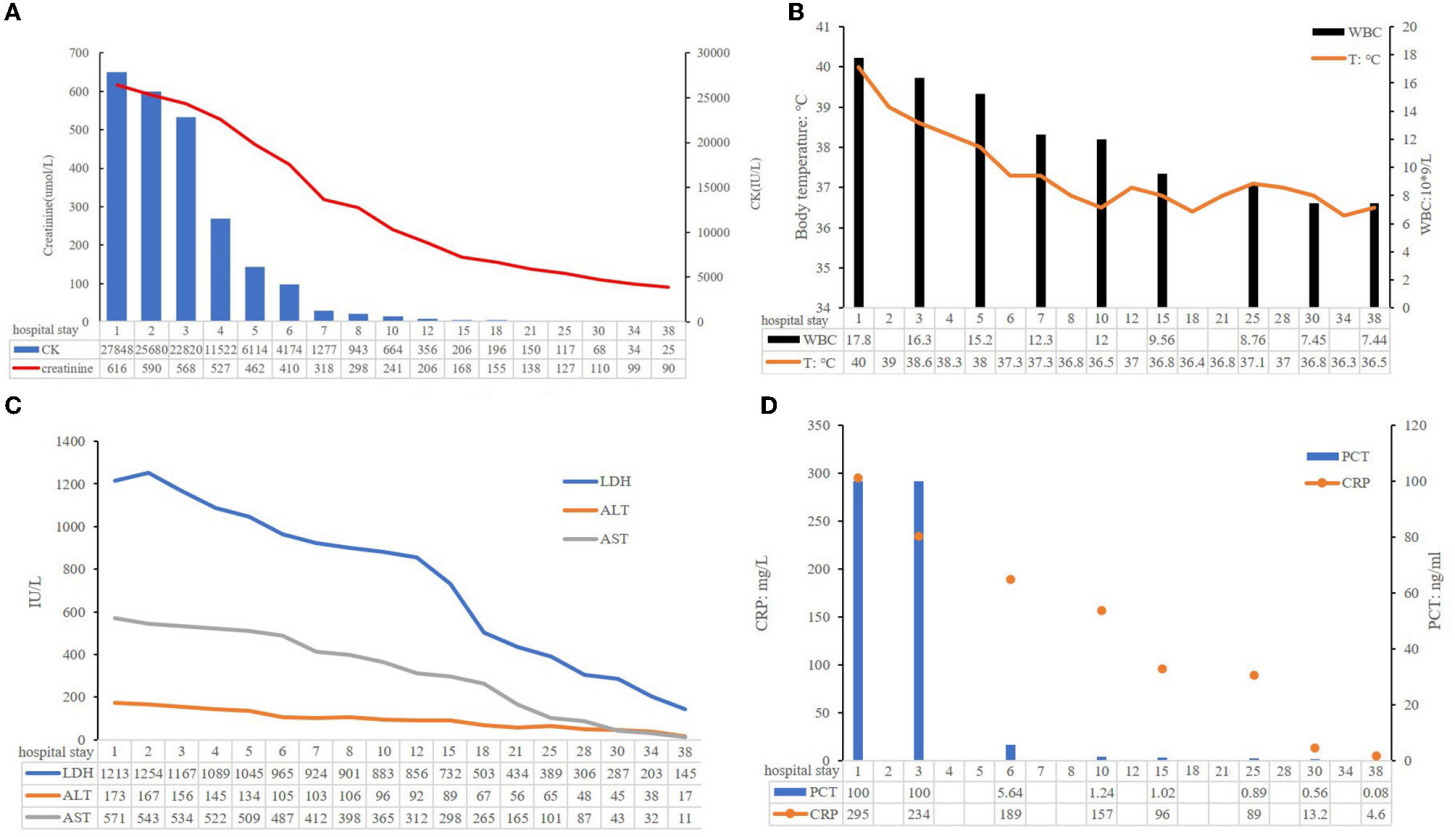
Some clinical indicators of this patient during hospitalization. **(A)** Creatinine and CK levels; **(B)** ALT, AST, and LDH levels; **(C)** WBC and body temperature; **(D)** PCT, CRP levels.

**Figure 4:**
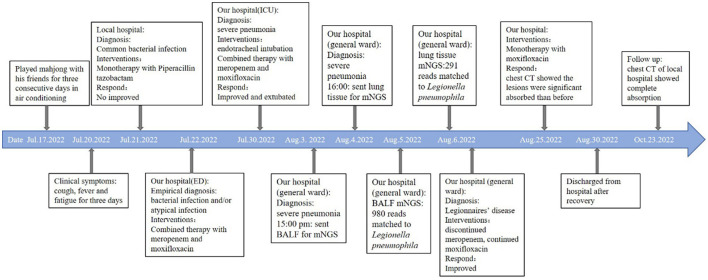
Timeline of the patient with relevant data of the episodes and interventions.

In total, 147,673,559 clean sequence reads were obtained in the BALF. When human host reads were excluded, 1,021 sequence reads were identified as *Legionella* at the genus level, 980 of which matched *L. pneumophila* at the species level. mNGS of the lung tissue yielded 131,264,566 clean sequence reads. When human host reads were excluded, 309 sequence reads were identified as *Legionella* at the genus level, 291 of which matched *L. pneumophila* at the species level (Figure 2). Experts in respiratory illness, microbiology, and radiology interpreted the results to identify potential etiological agents. Moxifloxacin monotherapy was used according to these results, and meropenem was weaned. Chest radiography showed significant improvement after 7 days, and the patient was extubated. After 15 days in the ICU, the patient was transferred to the general ward for treatment. Kidney function recovered gradually, and the frequency of hemodialysis changed from once a day to every other day and continued until day 15. The second anti-*Legionella* antibody test was positive 22 days after the onset of the disease. After 23 days of moxifloxacin treatment, chest CT revealed significant absorption of lesions (Figures 1C, D). He was discharged from the hospital after 38 days with normal creatinine and CK, ALT, AST, LDH, WBC count, PCT, and CRP levels (Figure 3). The timeline of patients with relevant data on episodes and interventions is presented in Figure 4.

## Discussion

*Legionella* is gram-negative rod-shaped bacteria ubiquitously found in fresh water environments and moist soil (6). *Legionella* pneumonia accounts for 2–15% of all community-acquired pneumonia (CAP) cases requiring hospitalization. It is the second most common cause of serious pneumonia and requires ICU admission (7). Legionnaire's disease is a severe form of pneumonia caused by *Legionella*; it can have extrapulmonary manifestations (8). Most cases are caused by *L. pneumophila*, whereas some are caused by *Legionella longbeachae*. Male sex (>50 years of age), smoking, and diabetes mellitus are the risk factors (9). Approximately 62% of the cases occur during summer and early autumn (10), which is related to the use of air conditioning. In this case, although the patient had no history of diabetes, his random blood glucose level was 24.0 mmol/L, and his glycosylated hemoglobin level was 13.3%. Therefore, the patient was diagnosed with type 2 diabetes. Generally, he had four risk factors: sex, age, smoking history, and type 2 diabetes. Before the symptoms appeared, he played Mahjong with his friends for 3 consecutive days on air conditioning. We suspected that Mahjong parlor was the source of the *L. pneumophila* infection.

No clinical manifestations unique to Legionnaire's disease were observed. The symptoms of Legionnaires' disease from most to least common include fever, cough, chills, dyspnea, neurological abnormalities, myalgia or arthralgia, diarrhea, chest pain, headache, and nausea or vomiting (10). Non-specific laboratory findings are common, such as elevated CK level, myoglobinuria, hyponatremia, microscopic hematuria, and leukocytosis (10). The rate of hyponatremia was 25.3% in a study of CAP (11). In Legionnaires' disease, the rate of hyponatremia was 44.4% (12). Hyponatremia has a negative impact on multiple outcomes, such as the need for mechanical ventilation and ICU care, the duration of hospital or ICU stay. In particular, hyponatremia adds more than 10,000 RMB to the cost of care (13). In studies of *Legionella*-related CAP, hyponatremia (< 133 mmol/L) was one of the strongest predictors (14, 15). Our patient had hyponatremia, elevated CK level, myoglobinuria, and leukocytosis, which were important reasons that the attending physician suspected *Legionella*-related CAP and administered moxifloxacin.

Furthermore, rhabdomyolysis is a syndrome caused by the breakdown and necrosis of muscle tissue and release of intracellular contents into the bloodstream (16). A diagnosis can be made when the serum CK level is >1,000 U/L (16). In adults, common causes are trauma and infection. The reported viruses and bacteria that can cause rhabdomyolysis include the following: influenza A and B, coxsackievirus, Epstein–Barr virus, primary human immunodeficiency virus, *Legionella* species, *Streptococcus pyogenes, Staphylococcus aureus* (*pyomyositis*), and *Clostridium* (17). Rhabdomyolysis caused by bacteria is associated with high mortality and morbidity: 57% of the cases lead to AKI, and 38% result in death (18). It can cause subacute- or acute-onset myalgia, transient muscle weakness, and dark tea- or cola-colored urine (16). Rhabdomyolysis induced by *Legionella* is rare. Prompt recognition is important for doctors to provide timely and appropriate treatment. In the present case, the patient was initially administered piperacillin-tazobactam monotherapy; however, no response was observed. Differential diagnoses of *S. pyogenes* and *S. aureus* can be excluded.

AKI refers to a sudden loss of excretory kidney function determined by increased serum creatinine levels and reduced urinary output, which can be caused by various factors (19). Infections and hypovolemic shock are the primary causes of AKI in low- and middle-income countries (19). In high-income countries, it mostly occurs in hospitalized older patients and is associated with sepsis, drug use, or invasive procedures (19). In non-traumatic rhabdomyolysis, AKI related to myoglobinuria is a serious complication. Patients who develop AKI have an increased mortality rate of 80% (20). Considering *Legionella* infection, the exact pathophysiology of rhabdomyolysis and AKI is poorly understood and is currently suspected to be endotoxin-mediated (21). It is thought to be induced by rhabdomyolysis; it is also thought to be induced by direct bacterial inoculation of the renal tissue (22). In our case, the CK level of the patient was 27,848 IU/L, he was anuric, with no more than 40 mL dark-tea urine in 24 h, and the synchronous serum creatinine was 616.00 μmol/L when he came to our hospital. In this patient, renal function was severely impaired in the initial stage of the disease, and it was difficult to determine whether AKI was indirectly associated with *L. pneumophila* via rhabdomyolysis or directly affected by *L. pneumophila*.

The triad of *Legionella* pneumonia, rhabdomyolysis, and AKI is an unusual syndrome that was first reported in 1992 (23) and is associated with high morbidity and mortality rates (up to 40%). We performed a literature search of PubMed using the keywords “*L. pneumophila*” and “rhabdomyolysis” and “acute kidney injury or acute renal injury” There have been 13 published case reports of rhabdomyolysis, renal failure, and Legionnaires' disease. The full text of one case report was unavailable; therefore, 12 case reports were available (Supplementary material). There were 11 men and 1 woman, with an average age of 53 years (range: 26–67). CK levels ranged from 1,103 to 600,000 IU/L, and the serum creatinine ranged from 2.1 to 11.05 mg/dL. All patients received antibiotic therapy with macrolides and/or fluoroquinolones. In total, 7 patients underwent dialysis, and 11 recovered; one female patient and one male patient died. Therefore, an early diagnosis is crucial. We conclude that the timely administration of potent antibacterial drugs and hemodialysis treatment led to the recovery of renal function. All diagnoses were based on antigen or antibody tests. Our patient is the first case of the triad of *Legionella* pneumonia, rhabdomyolysis, and AKI diagnosed using mNGS.

mNGS is an unbiased approach that can theoretically detect all pathogens in clinical samples and is particularly suitable for complicated infectious diseases, including viral, bacterial, fungal, and parasitic diseases (24). mNGS has a significantly better pathogen detection yield than other methods, especially for difficult-to-culture pathogens such as *Legionella* (25–28). Clinicians often find it difficult to distinguish *Legionella* pneumonia from other causes of pneumonia because of the lack of specific clinical manifestations. The gold standard for diagnosis is culture; however, it is now rarely used because it is very time-consuming. Sputum smear microscopy depends on the number of bacteria and the operator's skill. It is difficult to distinguish *Legionella* from other bacteria using microscopic morphology. *Legionella* does not grow on the standard medium used in microbiology laboratories, and a specific medium containing yeast extract and activated charcoal (buffered charcoal yeast extract, BCYE) is required (2). Anti-*Legionella* antibodies in most patients develop only approximately 3 weeks after disease onset, and anti-*Legionella* antibodies are not suitable for patients with severe diseases, such as Legionnaires' disease. In many countries, urinary antigen test is the primary diagnostic technique, although it is poorly sensitive to strains that are non-*L pneumophila* serogroup 1 or other species, including *L. longbeachae* (8). Urine antigens were not available at our hospital. In this case, before the patient came to our hospital, he had been administered piperacillin-tazobactam at a local hospital but still had a fever. The conventional method yielded negative results. After got the sample less than 48 h, the results of mNGS sent to physicians. For fungi, 88,625 reads mapping to *Candida* were detected in BALF. At the same time, no fungi were found in the libraries from the lung biopsy. *Candida* was more common in oral cavity and the upper respiratory tract, so we concluded that some contaminants may be introduced in the process. mNGS detected *Legionella* in both the BALF and lung tissues. Based on his medical history, clinical symptoms, physical signs, results of auxiliary examinations, and mNGS of both BALF and lung tissue, we confirmed the causative pathogen and discontinued meropenem. Thus, this approach also facilitates the use of targeted and efficacious antimicrobial therapies and avoids antibacterial resistance caused by abuse. Indeed, a large number of reads is associated with relative prolonged time and financial costs. Despite the high cost, some severe patients will benefit from the use of mNGS due to timely and targeted treatment. Sometimes, the total cost of patients using multiple pathogen cultures and tests even exceeds mNGS. Meanwhile, with the continuous progress of sequencing technology, the price of mNGS is gradually decreasing.

*Legionella* is susceptible to erythromycin, clarithromycin, azithromycin, levofloxacin (LEV), and moxifloxacin (MOX) in clinical practice (29). Many antibiotic-resistant clinical isolates of *L. pneumophila* have been identified (30). These isolates were mainly azithromycin-resistant. A meta-analysis concluded that the effectiveness of macrolides or respiratory fluoroquinolones did not reduce mortality among patients with *Legionella* pneumonia (31). Therefore, clinicians should select an antibiotic that is better tolerated and can provide coverage for concomitant infections. Patients with extrapulmonary involvement are often treated with fluoroquinolones (18). These three fluoroquinolones have similar minimum inhibitory concentrations against *L. pneumophila* (30). However, LEV requires dose adjustment according to renal function. Ciprofloxacin should be administered twice daily, which is inconvenient and also requires dose adjustments according to renal function. MOX is an imported drug with proven efficacy and does not require dose adjustment in patients with impaired renal function; therefore, we chose MOX. The current antibiotic treatment strategy entails a 7–10 days course for mild cases and a 21 days course for severe cases, which can be adjusted according to the patient's clinical response. In our case, due to severe illness, we administered MOX for 30 days, including 23 days of intravenous administration and 7 days of oral treatment. Eventually, the patient recovered completely.

This case illustrates the potential for severe rhabdomyolysis in a patient with *Legionella* pneumonia. It is believed that the rapid initiation of precise antimicrobial treatment and early substitution of renal function resulted in good outcomes. mNGS can assist in diagnosing infections caused by difficult-to-culture pathogens early, such as *Legionella*, especially in resource-limited settings where specific assays, such as the urine antigen of *Legionella*, are not accessible.

## Data availability statement

The datasets presented in this study can be found in online repositories. The names of the repository/repositories and accession number(s) can be found below: https://www.ncbi.nlm.nih.gov/ and PRJNA951206.

## Ethics statement

Ethical review and approval was not required for the study on human participants in accordance with the local legislation and institutional requirements. The patients/participants provided their written informed consent to participate in this study. Written informed consent was obtained from the participant/patient(s) for the publication of this case report.

## Author contributions

RD and YF collected and interpreted the data. RD drafted the manuscript. All authors contributed to manuscript revision, read, and approved the submitted version.

## References

[1] Gonçalves IG Simões LC Simões M. Legionella pneumophila. Trends Microbiol. (2021) 29:860–1. 10.1016/j.tim.2021.04.005 33994277

[2] Viasus D Gaia V Manzur-Barbur C Carratala J. Legionnaires' disease: update on diagnosis and treatment. Infect Dis Ther. (2022) 11:973–86. 10.1007/s40121-022-00635-7 35505000 PMC9124264

[3] Simner PJ Miller S Carroll KC. Understanding the promises and hurdles of metagenomic next-generation sequencing as a diagnostic tool for infectious diseases. Clin Infect Dis. (2018) 66:778–88. 10.1093/cid/cix881 29040428 PMC7108102

[4] He D Quan M Zhong H Chen Z Wang X He F. Emergomyces orientalis emergomycosis diagnosed by metagenomic next-generation sequencing. Emerg Infect Dis. (2021) 27:2740–2. 10.3201/eid2710.210769 34546163 PMC8462323

[5] Miao Q Ma Y Wang Q Pan J Zhang Y Jin W. Microbiological diagnostic performance of metagenomic next-generation sequencing when applied to clinical practice. Clin Infect Dis. (2018) 67:S231–40. 10.1093/cid/ciy693 30423048

[6] Zhan X-Y Yang J-L Sun H Zhou X Qian Y-C Huang K. Presence of viable, clinically relevant *legionella* bacteria in environmental water and soil sources of China. Microbiol Spectr. (2022) 10:e0114021. 10.1128/spectrum.01140-21 35438512 PMC9241679

[7] Seegobin K Maharaj S Baldeo C Downes JP Reddy P. Legionnaires' disease complicated with rhabdomyolysis and acute kidney injury in an AIDS patient. Case Rep Infect Dis. (2017) 2017:1–5. 10.1155/2017/8051096 29109879 PMC5646314

[8] Phin N Parry-Ford F Harrison T Stagg HR Zhang N Kumar K. Epidemiology and clinical management of Legionnaires' disease. Lancet Infect Dis. (2014) 14:1011–21. 10.1016/s1473-3099(14)70713-3 24970283

[9] Burillo A Pedro-Botet ML Bouza E. Microbiology and epidemiology of Legionnaire's disease. Infect Dis Clin North Am. (2017) 31:7–27. 10.1016/j.idc.2016.10.002 28159177

[10] Cunha BA Burillo A Bouza E. Legionnaires' disease. Lancet. (2016) 387:376–85. 10.1016/S0140-6736(15)60078-2 26231463

[11] Tokgöz Akyil F Akyil M Çoban Agca M Güngör A Ozantürk E Sögüt G. Hyponatremia prolongs hospital stay and hypernatremia better predicts mortality than hyponatremia in hospitalized patients with community-acquired pneumonia. Tuberk Toraks. (2019) 67:239–47. 10.5578/tt.68779 32050865

[12] Schuetz P Haubitz S Christ-Crain M Albrich WC Zimmerli W Mueller B. Hyponatremia and anti-diuretic hormone in Legionnaires' disease. BMC Infect Dis. (2013) 13:585. 10.1186/1471-2334-13-585 24330484 PMC3880094

[13] Zilberberg MD Exuzides A Spalding J Foreman A Jones AG Colby C. Hyponatremia and hospital outcomes among patients with pneumonia: a retrospective cohort study. BMC Pulm Med. (2008) 8:16. 10.1186/1471-2466-8-16 18710521 PMC2531075

[14] Beekman R Duijkers RR Snijders DD van der Eerden MM Kross MM Boersma WWG. Validating a clinical prediction score for *Legionella*-related community acquired pneumonia. BMC Infect Dis. (2022) 22:442. 10.1186/s12879-022-07433-z 35534798 PMC9081661

[15] Miyashita N Horita N Higa F Aoki Y Kikuchi T Seki M. Validation of a diagnostic score model for the prediction of *Legionella pneumophila* pneumonia. J Infect Chemother. (2019) 25:407–12. 10.1016/j.jiac.2019.03.009 30935766

[16] Cabral BMI Edding SN Portocarrero JP Lerma EV. Rhabdomyolysis. Dis Mon. (2020) 66:101015. 10.1016/j.disamonth.2020.101015 32532456

[17] Bosch X Poch E Grau JM. Rhabdomyolysis and acute kidney injury. N Engl J Med. (2009) 361:62–72. 10.1056/NEJMra0801327 19571284

[18] Kao AS Herath CJ Ismail R Hettiarachchi ME. The triad of Legionnaires' disease, rhabdomyolysis, acute kidney injury: a case report. Am J Case Rep. (2022) 23:e936264. 10.12659/AJCR.936264 35655418 PMC9171840

[19] Kellum JA Romagnani P Ashuntantang G Ronco C Zarbock A Anders H-J. Acute kidney injury. Nat Rev Dis Primers. (2021) 7:52. 10.1038/s41572-021-00284-z 34267223

[20] Chatzizisis YS Misirli G Hatzitolios AI Giannoglou GD. The syndrome of rhabdomyolysis: complications and treatment. Eur J Intern Med. (2008) 19:568–74. 10.1016/j.ejim.2007.06.037 19046720

[21] Prasanna A Palmer J Wang S. Legionnaire's disease presenting with the Legionella triad (pneumonia, rhabdomyolysis, renal failure) and cardiac complications. Cureus. (2022) 14:e26056. 10.7759/cureus.26056 35865426 PMC9289647

[22] Soni AJ Peter A. Established association of legionella with rhabdomyolysis and renal failure: a review of the literature. Respir Med Case Rep. (2019) 28:100962. 10.1016/j.rmcr.2019.100962 31720209 PMC6838801

[23] Shah A Check F Baskin S Reyman T Menard R. Legionnaires' disease and acute renal failure: case report and review. Clin Infect Dis. (1992) 14:204–7. 10.1093/clinids/14.1.204 1571431

[24] Goldberg B Sichtig H Geyer C Ledeboer N Weinstock GM. Making the leap from research laboratory to clinic: challenges and opportunities for next-generation sequencing in infectious disease diagnostics. mBio. (2015) 6:e01888-15. 10.1128/mBio.01888-15 26646014 PMC4669390

[25] Huang Y Ma Y Miao Q Pan J Hu B Gong Y. Arthritis caused by *Legionella micdadei* and *Staphylococcus aureus*: metagenomic next-generation sequencing provides a rapid and accurate access to diagnosis and surveillance. Ann Transl Med. (2019) 7:589. 10.21037/atm.2019.09.81 31807570 PMC6861802

[26] Yi H Fang J Huang J Liu B Qu J Zhou M. *Legionella pneumophila* as cause of severe community-acquired pneumonia, China. Emerg Infect Dis. (2020) 26:160–2. 10.3201/eid2601.190655 31855541 PMC6924908

[27] Wang Y Dai Y Lu H Chang W Ma F Wang Z. Case report: metagenomic next-generation sequencing in diagnosis of *Legionella pneumophila* pneumonia in a patient after umbilical cord blood stem cell transplantation. Front Med. (2021) 8:643473. 10.3389/fmed.2021.643473 34179036 PMC8232522

[28] Yue R Wu X Li T Chang L Huang X Pan L. Early detection of *Legionella pneumophila* and Aspergillus by mNGS in a critically ill patient with *Legionella* pneumonia after extracorporeal membrane oxygenation treatment: case report and literature review. Front Med. (2021) 8:686512. 10.3389/fmed.2021.686512 34277662 PMC8277993

[29] Niu S Zhao L. Metagenomic next-generation sequencing clinches the diagnosis of *Legionella* pneumonia in a patient with acute myeloid leukemia: a case report and literature review. Front Cell Infect Microbiol. (2022) 12:924597. 10.3389/fcimb.2022.924597 36478673 PMC9720252

[30] Yang JL Sun H Zhou X Yang M Zhan XY. Antimicrobial susceptibility profiles and tentative epidemiological cutoff values of *Legionella pneumophila* from environmental water and soil sources in China. Front Microbiol. (2022) 13:924709. 10.3389/fmicb.2022.924709 36312931 PMC9597688

[31] Jasper AS Musuuza JS Tischendorf JS Stevens VW Gamage SD Osman F. Are fluoroquinolones or macrolides better for treating *Legionella* pneumonia? A systematic review and meta-analysis. Clin Infect Dis. (2021) 72:1979–89. 10.1093/cid/ciaa441 32296816 PMC8315122

