# Supplementary material for: Metagenomic next-generation sequencing confirms the diagnosis of Legionella pneumonia with rhabdomyolysis and acute kidney injury in a limited resource area: a case report and review
Source: Front Public Health. 2023 May 9;11:1145733. doi: 10.3389/fpubh.2023.1145733 (PMC10205016; doi:10.3389/fpubh.2023.1145733)
Supplement: Supplementary file 1 [file Table_1.doc]

| Author, year | Gender | Age | Symptom | Risk factors/  underlying disease | CK  (IU/L) | Cr  (mg/dL) | Diagnosis method | Type | Antibacterial agents | Hemodialysis | ICU | Mechanical ventilation | Outcome |
| --- | --- | --- | --- | --- | --- | --- | --- | --- | --- | --- | --- | --- | --- |
| Masanori Abe, 2008(1) | man | 56 | general muscular weakness | alcoholic | 8,750 | 4.65 | Urine antigen | L. pneumophila serogroup 1 | EM, RFP, CPFX | Yes | / | Yes | died |
| Karan Seegobin, 2017(2) | man | 51 | fever, shortness of breath, nonproductive cough, headache and reduced appetite. | HIV, COPD, and hypertension | 51,092 | 6.90 | Urine antigen | L. pneumophila serogroup 1 | azithromycin and LEV | Yes | Yes | Yes | recovery |
| C Laivier,  2019(3) | man | 53 | diarrhea, weakness, abdominal pain, lack of appetite, dry cough, and dyspnea on exertion | smoking and chronic alcoholism | 96,012 | 4.48 | Urine antigen | L. pneumophila serogroup 1 | Cefuroxime and clarithromycin，changed: clarithromycin | Yes | Yes | Yes | recovery |
| Joshua McConkey, 2006(4) | man | 56 | diarrhea, weakness, shortness of breath, fatigue | alcoholic drinks, tobacco use | 5,141 | 1.90 | Urine antigen | L. pneumophila serogroup 1 | cefotaxime, acyclovir, vancomycin changed：moxifloxacin and piperacillin/tazobactam | NO | Yes | NO | recovery |
| Bayu Sutarjono,  2019(5) | man | 50 | fevers, sweating, shortness of breath, dyspnea, and mild  cough with no sputum production, rhinorrhea, diarrhea, weakness | tobacco use | >160,000 | 2.60 | Urine antigen | L. pneumophila serogroup 1 | vancomycin and cefepime changed: azithromycin | NO | Yes | NO | recovery |
| A Shah,  1992(6) | man | 26 | nonproductive cough, fever, chills, myalgia, vomiting, and diarrhea. | no | 8,520 | 9.6 | indirect immunofluorescence of the biopsy specimen | Legionella | EM | Yes | / | / | recovery |
| Author, year | Gender | Age | Symptom | Risk factors/  underlying disease | CK  (IU/L) | Cr  (mg/dL) | Diagnosis method | Type | Antibacterial agents | Hemodialysis | ICU | Mechanical ventilation | Outcome |
| Bruno Sposato,  2003(7) | woman | 61 | fever, severe respiratory failure, oliguria | / | 16,738 | 2.1 | anti- legionella IgG and IgM, Urine antigen | L. pneumophila serogroup 1 | clarithromycin and RFP | Yes | / | / | died |
| Jose Orsini,  2020(8) | man | 65 | dyspnea, generalized malaise and weakness, decreased oral intake | systemic arterial hypertension, dyslipidemia, and COPD resulting from long-standing nicotine use | 1,103 | 7.7 | Urine antigen | Legionella and Streptococcus pneumoniae | ceftriaxone and azithromycin | NO | Yes | Yes | recovery |
| Arnaud Ba,  2016(9) | man | 46 | diffuse abdominal pain, nausea, anorexia and rectal bleeding, constipation, dyspnea, fatigue and generalized myalgia | hemorrhoids | 600,000 | 9.69 | Urinary soluble Ag | L. pneumophila serogroup 1 | (anti-biotic therapy) | Yes | Yes | NO | recovery |
| Akinobu Kawai,  2004(10) | man | 66 | pyrexia | / | / | / | Urine antigen | L. pneumophila serogroup 1 | meropenem and EM  changed: CPFX | NO | NO | NO | recovery |
| N Matsumoto,  2000(11) | man | 67 | fever, chills, weakness, cough | / | 5,068 | 3.8 | indirect fluorescent antibody testing | L. pneumophila serogroup 1 | EM | Yes | / | / | recovery |
| J Labidi,  2006(12) | man | 39 | fever, dyspnea and productive cough with purulent sputum | no | 3,276 | 11.05 | positive serology | L. pneumophila | LEV and erythromycin | NO | NO | NO | recovery |

Abbreviations: M, erythromycin; CPFX, ciprofloxacin; LEV, levofloxacin; RFP, rifampicin.

**Reference**

1. Abe M, Kaizu K, Matsumoto K. Clinical evaluation of pneumonia-associated rhabdomyolysis with acute renal failure. Ther Apher Dial. 2008;12(2):171-175. doi:10.1111/j.1744-9987.2008.00565.x.

2. Seegobin K, Maharaj S, Baldeo C, Downes JP, Reddy P. Legionnaires' Disease Complicated with Rhabdomyolysis and Acute Kidney Injury in an AIDS Patient. Case Rep Infect Dis. 2017;2017:8051096. doi:10.1155/2017/8051096.

3. Laivier C, Bleuze MO, Hantson P, Devos J. Extreme Rhabdomyolysis, Acute Renal Failure, and Protracted Ileus in a Case of Legionella Pneumonia. Case Rep Crit Care. 2019;2019:3472627. doi:10.1155/2019/3472627.

4. McConkey J, Obeius M, Valentini J, Beeson MS. Legionella pneumonia presenting with rhabdomyolysis and acute renal failure: a case report. J Emerg Med. 2006;30(4):389-392. doi: 10.1016/j.jemermed.2005.07.016.

5. Sutarjono B, Alexis J, Sachidanandam JC. pneumonia complicated by rhabdomyolysis. BMJ Case Rep. 2019;12(6):e229243..doi:10.1136/bcr-2019-229243.

6. Shah A, Check F, Baskin S, Reyman T, Menard R. Legionnaires' disease and acute renal failure: case report and review. Clin Infect Dis. 1992;14(1):204-207. doi: 10.1093/clinids/14.1.204.

7. Sposato B, Mariotta S, Ricci A, Lucantoni G, Schmid G. [Legionnaire's pneumonia with rhabdomyolysis and acute renal failure. A case report]. Recenti Prog Med. 2003;94(9):391-394.

8. Orsini J, Frawley BJ, Gawlak H, Gooch R, Escovar J. Severe Sepsis With Septic Shock as a Consequence of a Severe Community-Acquired Pneumonia Resulting From a Combined Legionella pneumophila and Streptococcus pneumoniae Infection. Cureus. 2020;12(10):e10966. doi:10.7759/cureus.10966.

9. Bac A, Ramadan AS, Youatou P, Mols P, Cerf D, Ngatchou W. [Legionnaires' disease complicated by rhabdomyolysis and acute renal failure: about a case]. Pan Afr Med J. 2016;24:126. doi: 10.11604/pamj.2016.24.126.8536. eCollection 2016.

10. Kawai A, Nakajima H, Sawaguchi H, Touda Y, Nakajima S. [Surviving case of Legionella pneumonia showing a high level of serum KL-6 and complicated with rhabdomyolysis]. Nihon Kokyuki Gakkai Zasshi. 2004;42(8):737-742.

11. Matsumoto N, Mukae H, Yamashita S, Iiboshi H, Hiratsuka T, Katoh S, Matsukura S. [A case of severe Legionnaires' disease complicated by rhabdomyolysis, acute renal failure, liver dysfunction and encephalopathy]. Kansenshogaku Zasshi. 2000;74(11):989-993. doi: 10.11150/kansenshogakuzasshi1970.74.989.

12. Labidi J, Fdhila W, Battikh R, Ellouze S, Ben Abdelhafidh N, Louzir B, M'Sadek F, Othmani S. [Legionnaire's disease complicated by acute renal failure due to rhabdomyolosis: a case report]. Med Mal Infect. 2006;36(9):476-478. doi: 10.1016/j.medmal.2006.07.005. Epub 2006 Oct 4.
